# Supplementary material for: Are digital social media campaigns the key to raise stroke awareness in low-and middle-income countries? A study of feasibility and cost-effectiveness in Nepal
Source: PLoS One. 2023 Sep 8;18(9):e0291392. doi: 10.1371/journal.pone.0291392 (PMC10490866; doi:10.1371/journal.pone.0291392)
Supplement: Attachment — Submitted filename: 20230423_Response to reviewers.docx [file pone.0291392.s001.docx]

**Response to reviewers: Are digital social media campaigns the key to raise stroke awareness in low-and middle-income countries? A study of feasibility and cost-effectiveness in Nepal.**

|  | **Journal requirements** | Author's response |
| --- | --- | --- |
| 1. | 1. Please ensure that your manuscript meets PLOS ONE's style requirements, including those for file naming. | The style of the manuscript was revised and meets PLOS One’s requirements. |
| 2. | Please state what role the funders took in the study. | The funding statement was revised in the cover letter as follows: "The funders had no role in study design, data collection and analysis, decision to publish, or preparation of the manuscript." |
| 3. | Please complete your Competing Interests | The conflict of interest was added in the cover letter and the online submission form as follows: “The authors have declared that no competing interests exist.” |
| 4. | Should your manuscript be accepted for publication, we will hold it until you provide the relevant accession numbers or DOIs necessary to access your data. If you wish to make changes to your Data Availability statement, please describe these changes in your cover letter and we will update your Data Availability statement to reflect the information you provide. | Research data are publicly available on https://tunkl.de/research/nsp-pa/. Access to the Social Media accounts (Twitter, Meta, TikTok) to retrieve the original data can be made available by request“. |
| 5. | Please amend the methods section and ethics statement of the manuscript to explicitly state that the patient/participant has provided consent for publication: “The individual in this manuscript has given written informed consent (as outlined in PLOS consent form) to publish these case details”. | The images of human subjects do not depict patients or study participants, but the official campaign images of the World Stroke Campaign. |
|  | **Reviewer #1** |  |
| 1. | Minor point - in the second sentence of the background, it states that lack of stroke awareness hinders the benefits of stroke treatment. I would suggest that lack of awareness does not impact the magnitude of benefit of the treatment. Rather, it hinders the effective provision of stroke treatment (meaning it reaches less people, not that it's less effective for the patients it reaches). | The abstract was modified according to your suggestions (p.2, line 26: *"...the lack of public stroke awareness especially in low- and middle-income countries (LMICs) such as Nepal severely hinders the effective provision of stroke care."* |
| 2. | In the 2nd paragraph, it would be helpful to provide a bit of information about the studies that "allow one to assume" that stroke knowledge is low in Nepal. | We appreciate your feedback on the introduction and elaborated the section about prior studies in Nepal (p.4, ll. 56: "*In Nepal, recent studies demonstrated that knowledge on stroke in high-school students and in a rural population is insufficient[7, 8] with e.g. 55% of study participants believing in ayurvedic therapy to be effective in stroke care. The lack of awareness regarding stroke treatments and inadequate recognition of stroke symptoms have been identified as main factors contributing to delayed presentation at a tertiary care center[9] and therefore hampering the benefit of treatment.")* |
| 3. | The authors note that traditional mass media campaigns are often ineffective at improving health knowledge or behavior. Yet, they state that their hypothesis that a social media campaign would be more cost effective than a classical mass media campaign. I would expect that part of the reason for investigating social media campaigns is that they may also be more effective, but this is not included in the author's hypotheses. The authors also allude to reasons why it seems they think a social media campaign may be more effective (targets younger people more and younger people may be more impacted by health education campaigns, etc.). It would make the Introduction stronger if the authors specifically stated any hypotheses as to why a social media campaign may be effective compared to classical mass media campaigns that have been previously shown to have limited effectiveness. | We thank you for your comment. Accordingly, we modified the introduction and highlighted the assumed benefits of social media-based campaigns (p.4, ll. 72: "*In the last decade, social media has emerged as a platform with an enormous outreach with more than five billion people worldwide owning a smartphone[14]. By facilitating information sharing opportunities and community-building, social media has become invaluable in marketing and a promising approach in health education[15]. Especially young adults have expressed their interest in receiving health information via social media platforms[16] and a review found that social media even has the ability to facilitate mass communication, health education and knowledge translation in LMICs.")* |
| 4. | It would be interesting to note whether there were any trends in increased organic traffic to your social media accounts during or right after the paid advertisements? | We completely agree with you, that the association between paid advertisements and engagement to organic posts would be interesting to study. As our study design intended parallel playout of organic and paid-for posts, our data do not allow to answer your questions. |
| 5. | Page 17, line 319 "saturation of engagement" - please define | We reformulated the sentence to improve understandability: p.18, l. 340: “*We did not observe a decrease in engagement within the time course which leads to the assumption that we can potentially reach an even larger proportion of the general population if increasing the budget.”* |
| 6. | Page 19, line 340: Is this information on cost in the results section? I can't find it there. If not, would first introduce this in the Results section such that you are not introducing new data in the Discussion section. | We agree with your comment and added the information in the results section (p. 15, l. 303: "*TikTok required its own short video format, the costs for each video production were 20 EUR, which adds to a total of 1,130 EUR and a CPM of 7 EUR.")* |
| 7. | In the limitations section, the authors should address the limitations of their hypothesis that their social media campaign was so effective because of the "high quality" and "visually appealing" content. This may be, but users' experience was not assessed so there is no way to know this for certain. Depending on the structure of the planned qualitative survey, this could also be addressed in that work. | We put into perspective that the user’s experience yet needs to be assessed (p.22: ll. 440: *"While we assume the quality of the material had a positive effect on the engagement of users, the user’s experience should be addressed in a qualitative survey.")* |
| 8. | Minor error, page 21, line 408 - in the limitations section, it is written "but we CAN assume that the results are equally transferrable to other relevant health topics." Based on the remainder of the paragraph, I believe the authors meant to say "cannot" | Thank you for reading carefully, the mistake was corrected (p. 22, l. 434: *"... we cannot assume that the results are equally transferable to other relevant health topics*"). |
|  | **Reviewer #2** |  |
|  | I looked at twitter for the mentioned handle "@nepalstrokeproject" - but there is no such handle. Instead there is "@NepalStroke" handle which is named "Nepal Stroke Project". | We corrected the Twitter Handle in the manuscript, which is @NepalStroke (p. 7, l. 145) |
|  | This account has 83 followers and it follows 139 people/accounts. The accounts earliest post is on 1/2/2023. Initial tweets had less than 10 likes and got first 10 likes in April 2022 and next >10 like post was the photograph of WSC on 28/10/22. I am not able to verify the views of earlier posts but the recent posts have around 200-300 views. | We clarified in the manuscript, that Twitter was used for organic traffic only in contrast to paid-for advertisements on Facebook and Instagram. Further, we highlighted that Twitter was used solely for project-bound information. As correctly stated by the reviewer, Twitter did not reach high numbers of views and engagements, which we attribute to the fact that Twitter was used only for project-specific updates but not for promoting the World Stroke Day Campaign. (p. 7, ll. 148: "*While Facebook and Instragram were used to promote the World Stroke Day Campaign, Twitter was used exclusively for promoting project-specific information.";* p.9, ll. 193: *A predefined action plan combining organic traffic for Facebook, Instagram, Twitter and TikTok and paid-for advertisements on Facebook and Instagram was developed by a marketing expert.")* |
|  | At least in twitter- I couldn't see any major campaign slides or videos from the "@NepalStroke" handle. In the figure, the authors mention paid campaigns in May and June- but I am not able to see any post in May. Usually in twitter, it will show "promoted" for a paid campaign- but I am not able to see along with any posts. | We emphasized in the “limitations” section that Twitter was not comparable to Facebook and Instagram in terms of reach/ engagement, as posts were not promoted with paid advertisements (p.22, ll. 431: "*We used paid advertisement for Facebook and Instagram only, but our study excluded other relevant advertisement channels as YouTube, Google and TikTok as well as paid advertisement on Twitter or TikTok, which impedes a comparison between different channels.*") |
|  | The authors mention that there is no option of knowing "reach" in twitter - but there is an option of knowing "views" (times a tweet was seen on twitter) | As the reviewer correctly stated Twitter provides the number of “views”, which we mentioned in the paper as “impressions” according to Twitter’s definition of “view”. As Twitter counts the number of times a post is displayed, we can’t conclude that it was displayed to different people, which is the definition of “reach”. |
